# Mastering meta‐analysis in Microsoft Excel with MetaXL add‐in: A comprehensive tutorial and guide to meta‐analysis

**DOI:** 10.1111/jep.14138

**Published:** 2024-10-02

**Authors:** Ibrahim Elmakaty

**Affiliations:** ^1^ Department of Medical Education Hamad Medical Corporation Doha Qatar

**Keywords:** mean difference, meta‐analysis, MetaXL, network meta‐analysis, prevalence, ratios

## Abstract

**Rationale:**

Meta‐analysis, a powerful technique for combining effect estimates from multiple studies, enhances statistical power and precision. However, its adoption can be hindered by challenges in statistical interpretation and the complexity of specialized software. MetaXL, a freely available Microsoft Excel add‐in, aims to mitigate these barriers by providing comprehensive support and facilitating seamless integration of meta‐analytical results into research publications.

**Aims and Objectives:**

This tutorial illustrates the practical application of MetaXL for synthesizing meta‐analytical evidence, with a focus on common effect sizes and their presentation.

**Method:**

This paper reintroduce MetaXL's functions and provide concise explanations of common effect sizes employed in meta‐analysis. The tutorial delves into fundamental concepts such as the selection of appropriate effect sizes for pooling and the choice of meta‐analytical models. Eight illustrative examples are presented, incorporating diverse effect sizes and data formats, including scenarios involving incidence rate ratios, weighted and standardized mean differences, hazard ratios, and prevalence. Additionally, key concepts in network meta‐analysis are discussed, along with their implementation in MetaXL. MetaXL provides convenient access to data formatting templates tailored to various data types and effect sizes encountered in included studies.

**Results and Conclusion:**

This tutorial offers researchers, particularly those with limited resources, detailed explanations and insights into commonly used methodologies for pooling effect sizes. Furthermore, it introduces the new Excel functions that comes with the MetaXL add‐in. Accurate population of this function and adherence to the correct format are essential to ensure error‐free analyzes.

## 1 INTRODUCTION

The recent surge in scholarly publications has led to a bigger focus among researchers on the utility of systematic reviews. Systematic reviews constitute a research methodology wherein articles possessing similar attributes are systematically gathered, following strict protocols designed to mitigate biases.^1^ These protocols typically involve a thoroughly constructed search strategy, predefined eligibility criteria, and a careful assessment of risk of bias. Subsequently, each study meeting the inclusion criteria is subjected to an analysis of its outcomes of interest, which are then synthesized to yield novel insights based on a comprehensive summary of all included studies.

Frequently, these outcomes of interest are expressed as effect sizes, enabling their quantitative aggregation through a technique known as meta‐analysis. This process involves the pooling (e.g., combining) of effect sizes from individual studies to produce pooled effect sizes, thereby enabling researchers to derive new effect sizes with enhanced statistical power and precision.^2^

While conducting systematic reviews presents a manageable challenge, some researchers encounter difficulties when transitioning to meta‐analysis, consequently resulting in a reluctance among authors to advance their research to this higher level of analysis. Various factors contribute to this reluctance, including a deficiency in understanding statistical techniques, inadequate proficiency in coding, reliance on paid software, limited assistance from statisticians, the complexity of conducting analyzes, and difficulties in interpreting results.

MetaXL (EpiGear Int Pty Ltd.) is an add‐in specifically crafted for performing meta‐analyzes within the Microsoft Excel. The MetaXL version 5.3 software is freely accessible and can be obtained via the provided link: “https://www.epigear.com/index_files/metaxl.html”. The sole prerequisite for utilizing MetaXL is access to Microsoft Excel. This software offers comprehensive support for a diverse array of meta‐analysis techniques, presenting results in both tabular and graphical formats, thereby facilitating their integration into research articles with ease.

MetaXL was created by Jan Barendregt and Suhail Doi in response to two primary concerns with traditional meta‐analysis methods: heterogeneity between studies and publication bias. Traditional approaches, such as the random effects (RE) model, have limitations in handling heterogeneity. Specifically, the RE model if often criticized for underestimating the statistical error even when it is compared to the fixed‐effects (FE) model.^3^ This underestimation can lead to unjustifiable adjustments in study weights, thereby compromising the accuracy of the meta‐analysis. For this reason, MetaXL primary goal was to offer alternative meta‐analytical models like the inverse variance heterogeneity (IVhet), Quality effects (QE) models. Additionally, MetaXL offers an alternative to the traditional funnel plot for detecting publication bias. The Doi plot is designed to be more interpretable and effective in identifying potential publication bias.

**Figure 1:**
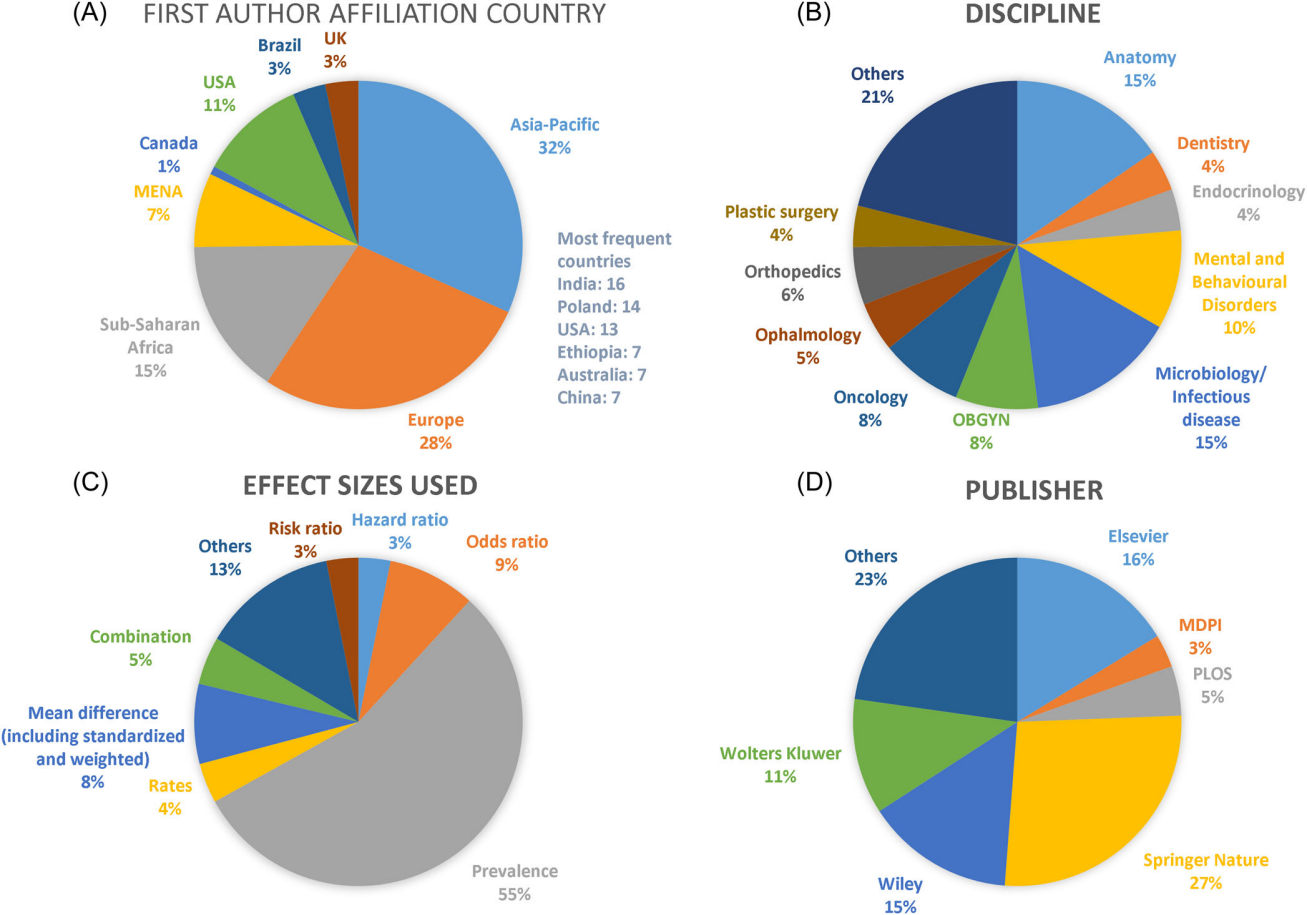
Displays a summary of the characteristics of articles on MetaXL retrieved from PubMed when searching for “MetaXL” on 2nd May 2024. (A) Depicts the country of affiliation of the first author. (B) Illustrates the discipline of the paper's topic. (C) Presents the types of effect sizes utilized. (D) Indicates the publishers of the papers. OBGYN, obstetrics and gynecology; UK, United Kingdom; USA, United States of America.

Despite its numerous advantages, MetaXL is currently not as widely utilized as other data analysis software tools. A rapid search conducted on PubMed on 2nd May 2024, using the term “MetaXL” yielded 139 references to published articles. Among these references,^4^ were excluded from consideration as they did not pertain to meta‐analysis (including 5 background papers on MetaXL, 8 papers on the MET/AXL signaling pathway, 2 duplicates, and 1 irrelevant paper). Of the remaining 123 articles, 70 (55%) utilized MetaXL solely for estimating prevalence. The frequent topics of estimation included infectious microorganisms and anatomical variations of normal surgical anatomy. Figure 1 provides a summary of the characteristics of these 123 meta‐analyzes that employed MetaXL.

Therefore, in this tutorial, I will illustrate the utilization of MetaXL for synthesizing meta‐analytical evidence, drawing examples from a group of selected published papers with modified data. The examples will primarily focus on the common types of effect sizes, and I will provide comprehensive explanations of their presentation and interpretations, along with foundational insights into the common methodologies employed for pooling effect sizes. The objective of this paper is to empower researchers with limited resources and knowledge of meta‐analysis, providing them with the essential skills needed to conduct meta‐analyzes using MetaXL.

## 2 MetaXL ADD‐ON OPTIONS

**Figure 2:**
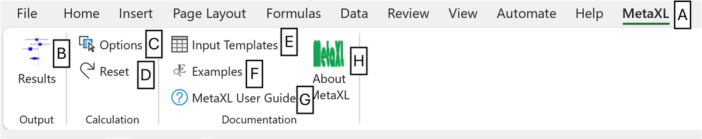
Illustrates the MetaXL tab along with its available options. Label A is the access point for available options. Label B directs to the results, where all analyzes done can be selected to view meta‐analysis results in forest plot graph and table formats. Label C offers options for adjusting forest plot graph and table outputs. Label D resets saved analysis results from memory, which is useful if the analysis function is updated or a new Excel sheet is opened (it does NOT reset the options modified in Label C options). Label E inputs suggested templates for required data in the correct format. Label F provides access to Excel files with pre‐typed examples for the analysis functions. Label G opens the user guide provided by the authors, and Label H provides information about the current version of MetaXL.

Once the MetaXL setup “.exe” file has been downloaded, the setup process is straightforward and swift. Upon completion, a new tab will appear in Excel, positioned to the left of the “Help” tab. Figure 2 illustrates the available options within MetaXL and their respective locations.

## 3 MetaXL FUNCTIONS

One of the most unique features of Excel is its vast variety of formulas and functions. The MetaXL add‐in provides access to new functions that we will use to perform the meta‐analysis. The main and most important function is “MAInputTable”. For this function to work, it requires four important inputs in the following order: (1) function name (Name), (2) function type code (IOType), (3) meta‐analysis model used for pooling (Method), and (4) a range of Excel cells with the extracted data to be used in the analysis (Table). Therefore, the function is typed in any empty Excel cell with the following format: =@MAInputTable(“Name”,“IOType”,“Method”,Table). Notice the absence of spaces between the function components, the starting with “=@” and the presence of double quotation marks for the first three options.

The function name is a unique identifier set by the researcher for each function. For instance, if we are conducting a meta‐analysis with two different outcomes of interest within the same Excel file, we need to assign distinct names to their functions. Any name will suffice, provided it is not overly long.

IOType comprises a set of predefined function type codes. Each code is selected based on the type of effect size to be generated and the format of the extracted data. We will explore some examples, while a comprehensive list of options is available in the user guide.

Regarding pooling methods, six options are accessible: Inverse variance (IV) (also known as fixed effects), RE, IVhet, QE, Mantel Haenszel (MH), and Peto method (Peto). MH and Peto are limited to binary data, hence we will refrain from using them in this tutorial. QE requires an additional input, as we will demonstrate in the examples.

Lastly, we need to specify the range of Excel cells containing the extracted data to be used in the meta‐analysis. Here is an example of what a function might look like: =@MAInputTable(“MyFirstFunction”,“NumRR”,“RE”,B6:H27).

In meta‐analysis, model selection are pivotal processes that should depend on the assumptions researchers are comfortable accepting with regards to the meta‐analytical model selected. The model selected should account for heterogeneity in its error estimation method. All meta‐analytic models, except for FE models (e.g., FE ignore heterogeneity), aim to incorporate this heterogeneity in their error estimation methods. Models such as the IVhet and QE models introduced by MetaXL specifically target the limitations of traditional RE models. Thus, the choice of a meta‐analysis model hinges on selecting one model that not only aligns with the initial assumptions by the model but also effectively accounts for heterogeneity in its error estimation, not by the level of heterogeneity across the included studies (e.g., selecting a cutoff in heterogeneity measurement to choose a model). Therefore, it is recommended to read the models assumption and explanation provided by the MetaXL user guide (Figure 2, label G).

## 4 POOLING RATIOS

### 4.1 Introduction to ratios

In meta‐analyzes, three important ratios are commonly pooled: odds ratio (OR), risk ratio (RR), and hazard ratio (HR). Typically, the choice among them depends on which ratios are provided by the included studies. These ratios can be pooled in two formats, which is crucial to understand before starting data extraction. The first format is the binary format, where the numbers of events and totals are extracted for both study arms. This format does not apply to HR, as it is a function of time. The second format involves extracting the ratio (effect size) along with its 95% confidence interval (CI).

Both formats offer advantages. The binary format enables the reconstruction of the ratios and their 95% CI in our analysis, providing reassurance. In the effect size and 95% CI format, some authors report adjusted values (adjusted for age, sex, etc.), which can be more accurate. Either format is acceptable.

### 4.2 Example 1: OR using binary data

#### 4.2.1 Example 1: Description and formatting

As this is the first example, we will go through all the details and interpretations. This example is demonstrated using data from one of my meta‐analyzes where we investigated the effect of a certain chemokine (extracted as either high or low expression in cells) potential in causing oral cancer metastasis to lymph nodes (extracted as either cancer cells are present in lymph nodes or not).^5^ A modified version of the data extracted is provided in Table 1, which be used in the analysis.

**Table 1:** Nodal metastasis numbers in each group based on chemokine expression.

Study	Chemokine expression	Nodal metastasis	No nodal metastasis
Tsuzuki	High chemokine	33	21
	Low chemokine	12	24
Patkin	High chemokine	53	7
	Low chemokine	9	15
Gonzalez‐Arriagada	High chemokine	21	21
	Low chemokine	14	20
Shang	High chemokine	31	25
	Low chemokine	8	21
Xu	High chemokine	27	27
	Low chemokine	3	18
Guo	High chemokine	28	18
	Low chemokine	8	24
Wang	High chemokine	20	21
	Low chemokine	4	15
Xia	High chemokine	22	17
	Low chemokine	5	16

**Figure 3:**
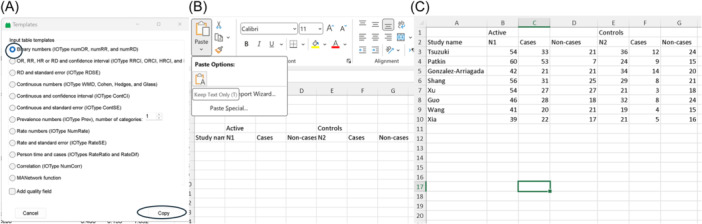
Illustrates the steps of data formatting. (A) Demonstrates how to select and copy the required template. (B) Illustrates how to paste it. (C) Shows the formatted data.

Although we have extracted the necessary data from the original studies, it is not in a format suitable for analysis in MetaXL; therefore, reformatting is necessary. Since we are using the binary format for pooling our effect size, we select “Input Templates” as shown in Figure 2 label E, and then choose the option for the binary format, as illustrated in Figure 3A. Next, we click on the “Copy” option (shown in Figure 3A). Now, the template is copied to the clipboard, and we can paste it anywhere in the Excel sheet, as depicted in Figure 3B. After that, we need to carefully transfer the data into the required format, as shown in Figure 3C (where “N” represents the total number of that group, “active” indicates high chemokine expression, and “cases” represent positive nodal metastasis).

#### 4.2.2 Example 1: Analysis

To conduct the meta‐analysis, we need to complete the template function outlined in Section 3: =@MAInputTable(“Name”,“IOType”,“Method”,Table). We will name the function as “Example 1”. The IOType for meta‐analysis of OR using binary data is “NumOR”. Table 2 displays all IOType codes relevant to pooling ratios.

**Table 2:** IOType codes relevant to pooling ratios.

IOType	Effect size	Choice in input templates	Required data
NumRR	Risk ratio	Binary numbers	Number of events in both groups
NumOR	Odds ratio	Binary numbers	Number of events in both groups
NumRD	Risk difference	Binary numbers	Number of events in both groups
RRCI	Risk ratio	OR, RR, HR, RD and confidence interval	Risk ratio and its 95% CI
ORCI	Odds ratio	OR, RR, HR, RD and confidence interval	Odds ratio and its 95% CI
HRCI	Hazard ratio	OR, RR, HR, RD and confidence interval	Hazard ratio and its 95% CI
RDCI	Risk difference	OR, RR, HR, RD and confidence interval	Risk difference and its 95% CI
RDSE	Risk difference	RD and standard error	Risk difference and its standard error

Abbreviation: CI, confidence interval.

**Figure 4:**
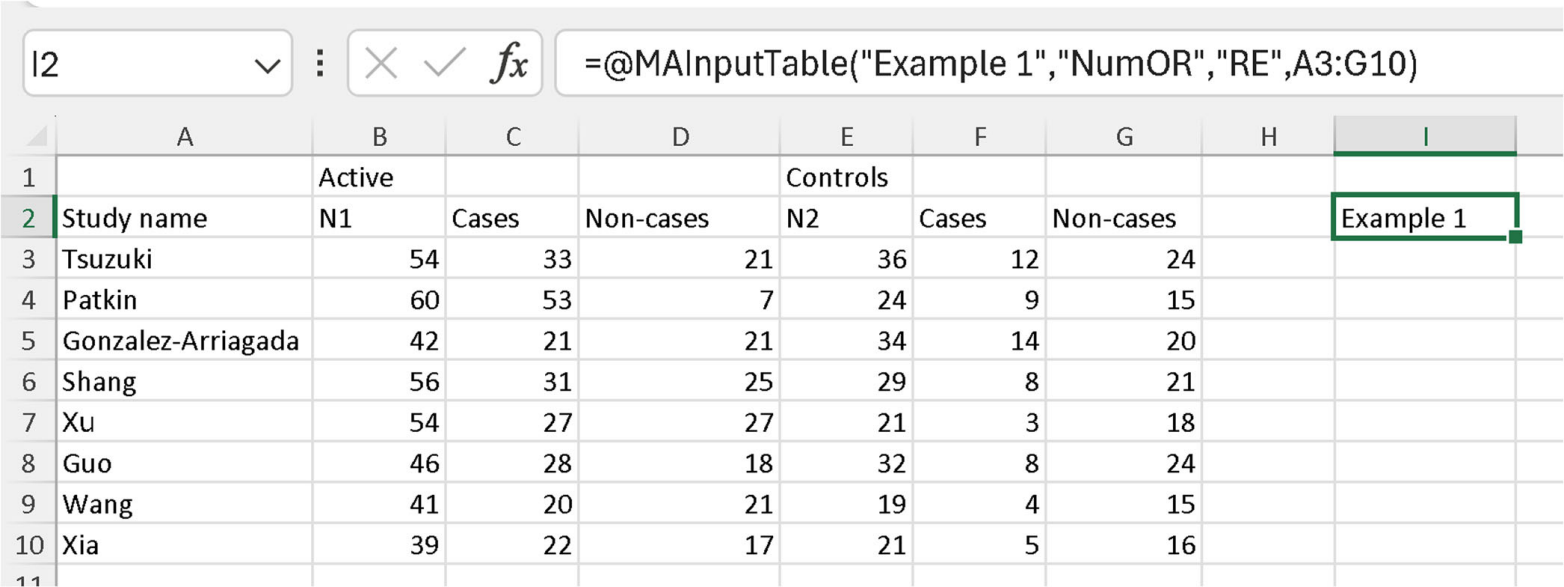
Shows the analysis function for “Example 1” meta‐analysis.

Since we have a sufficient number of studies without extracted quality scores, either “RE” or “IVhet” will suffice as a model. For this example, we will use “RE”. The table for our analysis will include selecting all the cells that we extracted from the included studies (do NOT select the template headers cells). Therefore, we will select the cells from A3 to G10, shown in Figure 3C. Now, all we have to do is select an empty cell and type our function, including the four mentioned data: =@MAInputTable(“Example 1”,“NumOR”,“RE”,A3:G10), as shown in Figure 4. The cell with the meta‐analysis now displays “Example 1”, as it is the name we chose for this function. Our meta‐analysis is now complete, and all we have to do is click on “Results” (Figure 2, label B) to view the results.

#### 4.2.3 Example 1: Reporting

**Figure 5:**
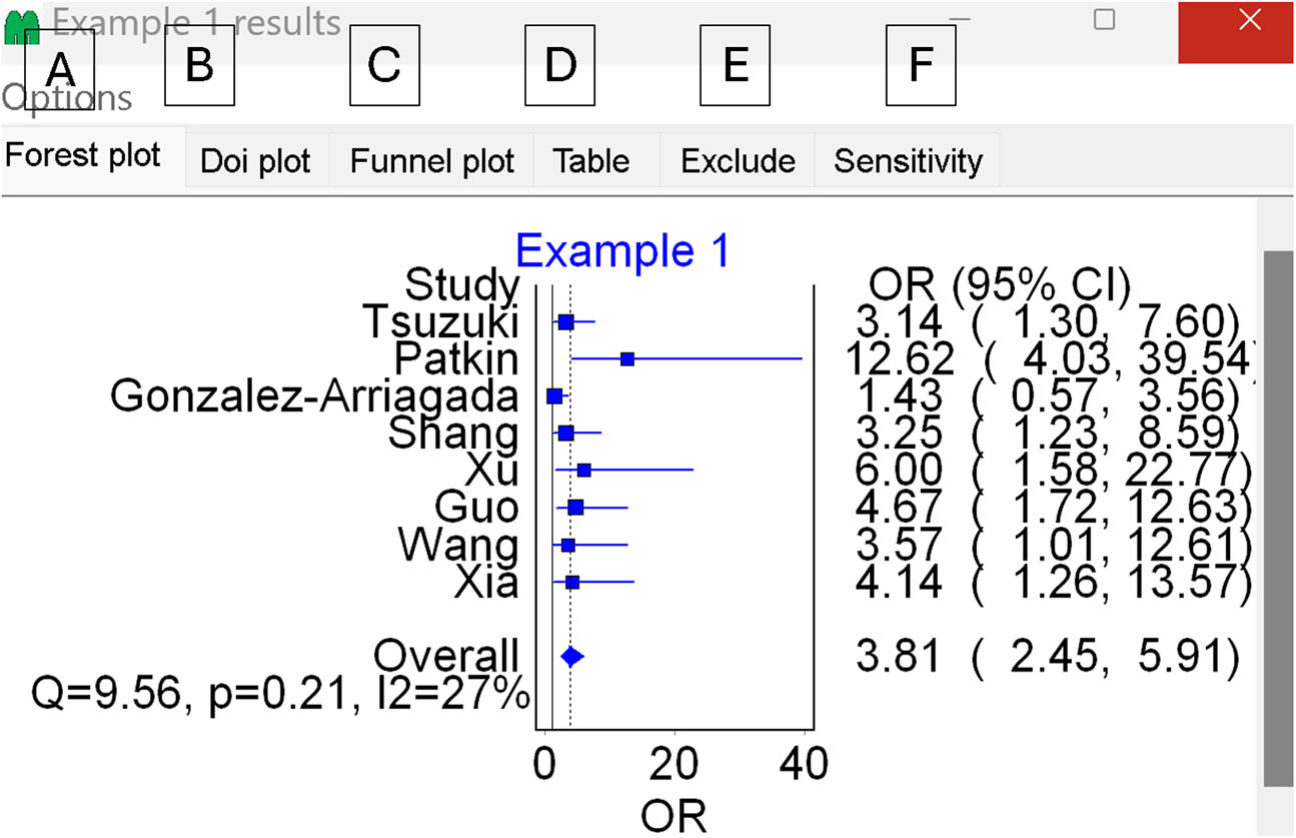
Displays the available options for the results. Label A represents the forest plot option, where we can utilize the figure to report our pooled effect size and its 95% confidence interval (CI), Cochran's Q, I‐squared, and each study's estimated effect size. Labels B and C indicate the options for the Doi plot and funnel plot, respectively, which are used for reporting publication bias. Label D provides the option for displaying the results in a table format rather than the forest plot in option A. Label E is for manually excluding studies to perform subgroup or sensitivity analysis. Label F offers the option of conducting a leave‐one‐out sensitivity analysis, which shows us the effect of removing the studies using the pooled effect size and heterogeneity.

Traditionally, in meta‐analysis, authors are expected to report the following: the final effect size and its 95% CI, the method used to assess heterogeneity, and the method used to evaluate publication bias, aligning with The preferred reporting items for systematic reviews and meta‐analyzes (PRISMA) guideline.^6^ A forest plot is also commonly used for reporting results. Additionally, there are occasions where performing subgroup or sensitivity analyzes becomes necessary. Subgroup analyzes involve examining findings within specific groups of participants within the sample.^7^ Sensitivity analyzes assess whether the conclusions, such as effect sizes and heterogeneity, remain consistent even when the data is handled differently than in the initial analytic plan.^7^ Bearing this in mind, Figure 5 displays the available results when clicking on the “Results” tab (Figure 2, label B).

**Figure 6:**
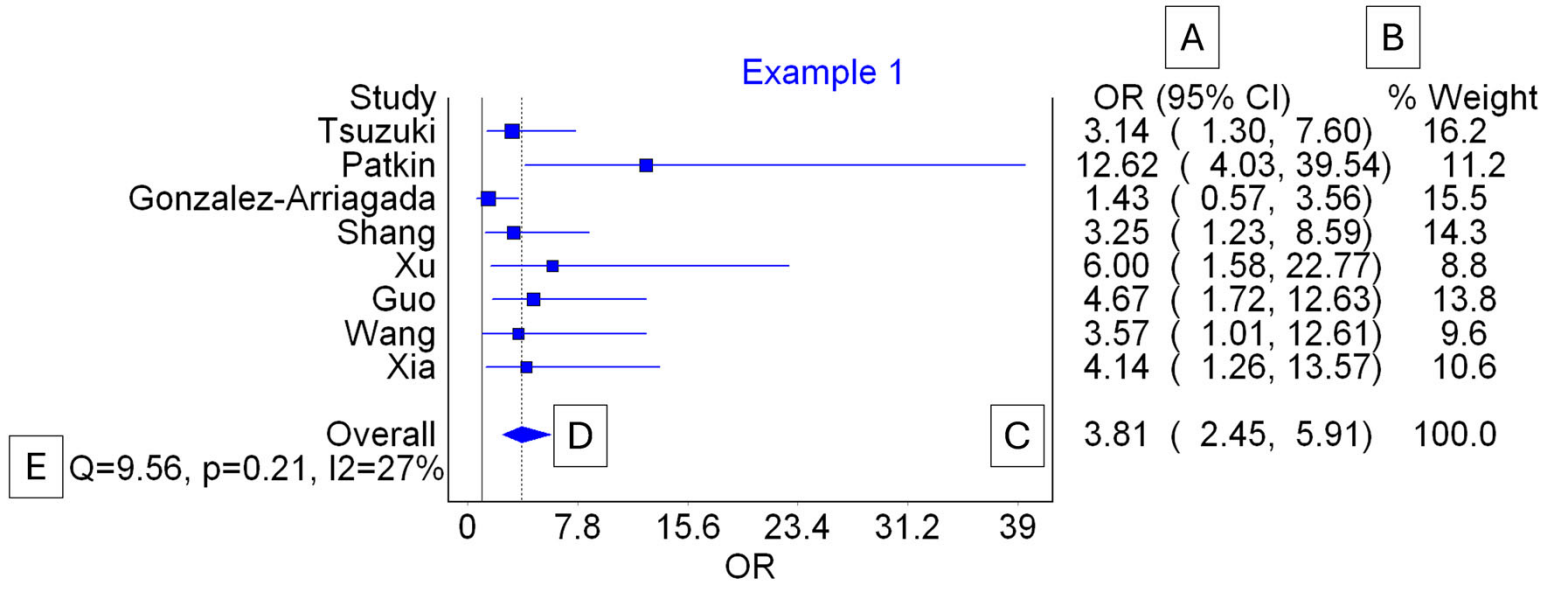
Displays the generated forest plot. Label A indicates where we can find the effect size generated for each study along with its confidence interval. Label B shows the weight of each study based on the model used (random effects in this example). Label C presents the pooled overall effect size and its confidence interval (CI), while label D represents this effect size on the graph using a diamond symbol. Label E indicates where heterogeneity measures are reported, including Cochran's Q, its *p*‐value, and I‐squared.

For this example, we will report the forest plot. It appears crumped and text is overlapping, therefore, we need to do some adjustment. We simply click on “Forest plot” option (Figure 5 label A). Then right click on the graph and select “Plot options”. In the Y‐axis section, we can increase the spacing and reduce the right margin to fix these issues and have a presentable forest plot. Plot options also give access to other options, like changes the colors. Once done with modifying the plot, close the window and right click on the graph again and select “Copy”. Now the graph is saved in the clipboard and can be inserted in the results section of our meta‐analysis. The final forest plot is shown in Figure 6 with labels to help in the understanding.

**Figure 7:**
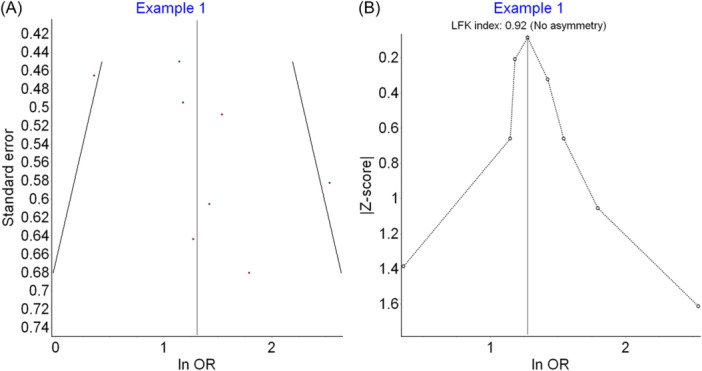
Shows the figures used for representing publication bias. (A) Shows the funnel plot. (B) Shows the Doi plot.

In our meta‐analysis manuscript, we must report that the pooled OR is 3.81 (95% CI: 2.45–5.91). For heterogeneity measurements, either reporting I‐squared = 27% or Cochran's Q *p*‐value of 0.21 will suffice. Similarly, either the funnel plot or the Doi plot can be reported to address publication bias. Figure 7 displays both the funnel and Doi plots for this example.

#### 4.2.4 Example 1: Interpretation

The pooled OR is 3.81 (95% CI: 2.45–5.91), indicating that the odds of developing nodal metastasis in samples with high chemokine expression are 3.81 times greater than those with low chemokine expression. This finding is significant both in terms of magnitude and because the CI does not cross the value one.

Heterogeneity poses a common challenge in meta‐analysis. It is expected that each study differs as it is conducted in a unique setting; however, there are broad causes for heterogeneity between the included studies: random error (which is always present) and systematic error. When a meta‐analysis reports significant heterogeneity, it implies that systematic error significantly influences the pooled estimates.^8^ Systematic error includes real differences between studies caused by factors such as varied treatment regimens, different patient populations, or the use of diverse diagnostic tests in accuracy studies.

Cochrane's *Q* test represents a weighted sum of squared differences between individual study effects and the overall effect size.^9^ Its *p*‐value is easily interpretable, with a value less than 0.05 indicating significant heterogeneity due to systematic differences between the included studies. The I‐squared statistic indicates the percentage of error attributable to real differences between studies (systematic error).^9^

In our example, Cochran's Q *p*‐value was 0.21, indicating no significant heterogeneity. The I‐squared value was 27%, suggesting that 27% of the differences were due to real systematic differences between the studies (e.g., 73% of the differences seen in the included studies are due to random error), indicating mild to moderate heterogeneity.

Publication bias occurs when statistically significant findings are more likely to be published than non‐significant ones (e.g., studies with higher effect sizes are published). Both the funnel plot and Doi plot are based on the effect size and its standard error. In the funnel plot (seen in Figure 7A), four studies are on each side of the graph, with one study outside the funnel on each side, indicating symmetrical distribution.^10^ The Doi plot in Figure 7B is also symmetrical, with an LFK index of 0.92, indicating no significant publication bias.^11^

### 4.3 Example 2: The addition of the QE model

The QE model assumes that studies of higher quality have a higher level of certainty in their findings and should therefore be given more weight in the analysis.^12^, ^13^, ^14^ Consequently, studies are ranked on a relative scale based on quality scores. Thus, the model requires the user to include quality scores. The quality score should be numerical and objective, which can be achieved by using a numerical quality assessment tool or by using any risk of bias tool with assigning a numerical value for each question. When all studies have similar quality, the QE model will generate a result similar to that of the IVhet model. Therefore, the QE model is most useful when the quality of the included studies differs significantly, which is often the case.

We will utilize the same data from Example 1 to illustrate the use of the QE model. In that example, some studies examined overall survival (OS) and recurrence of oral cancer (e.g., measurements that need follow‐up), requiring a retrospective cohort design, while others employed a cross‐sectional study design. Consequently, there was significant heterogeneity in terms of quality (it is well known that cohort studies are of higher quality compared to cross‐sectional), leading us to opt for the QE model.

**Figure 8:**
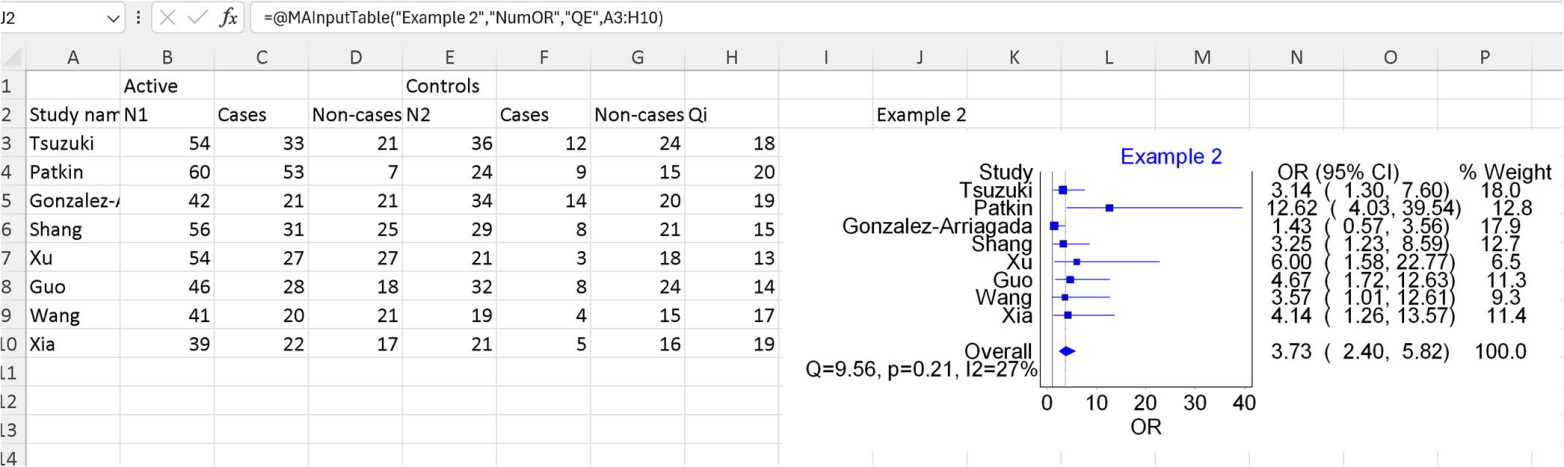
Showing the data and function used with the output forest plot for example 2.

Initially, we conducted a quality assessment using the methodological standards for epidemiological research (MASTER) scale,^15^ which enables assessment of the quality of different study designs. Subsequently, we followed the same steps outlined in Figure 3, selecting the option “Add quality field” as depicted in Figure 3A at the bottom right. Figure 8 displays the data used, Excel function, and output forest plot for this analysis. The remaining results to be reported and interpreted are similar to those illustrated in Example 1.

### 4.4 Example 3: Using HR with 95% CI

**Figure 9:**
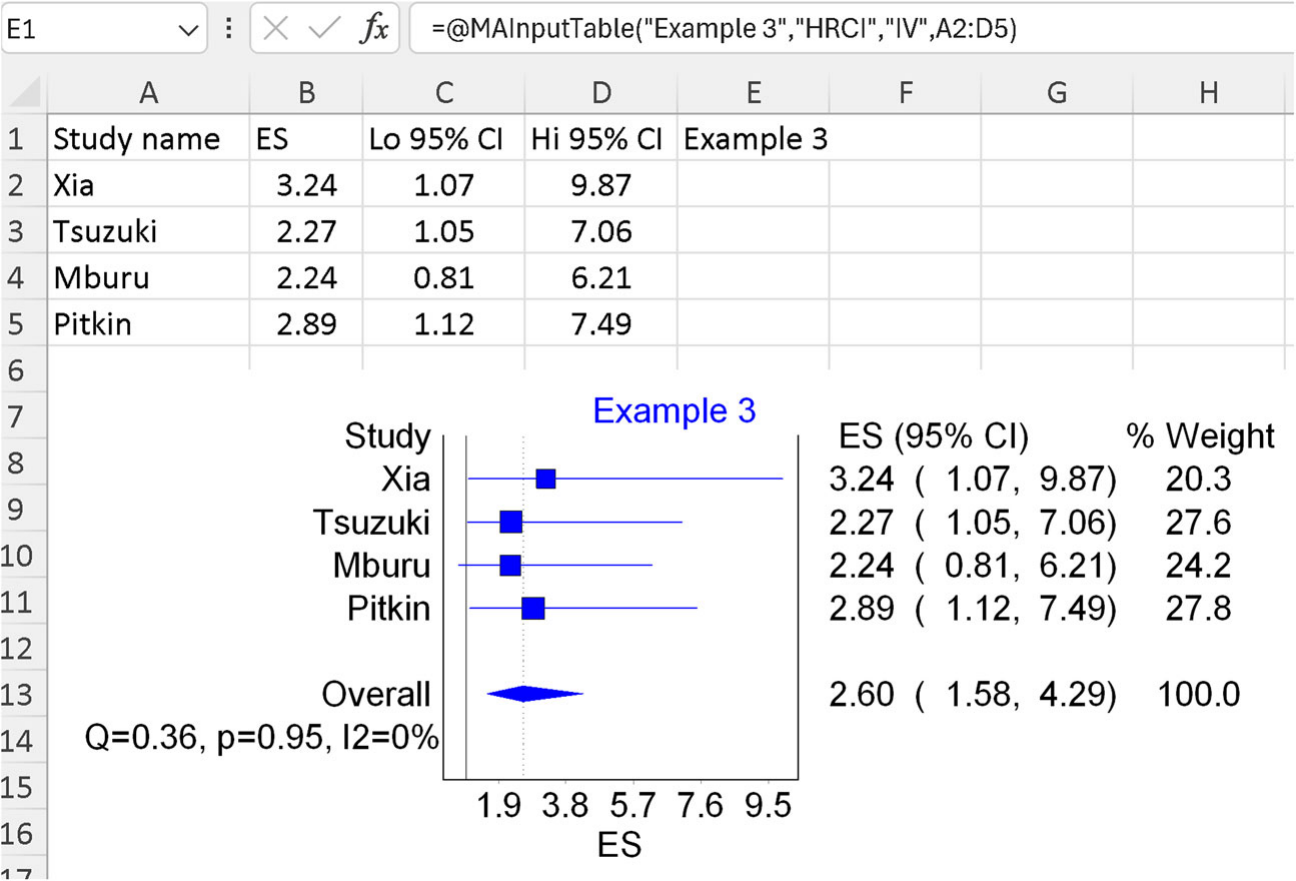
Shows the data and function used with the output forest plot for example 3.

In this third example, we will utilize data from the same study employed in example 1, focusing on examining OS concerning our chemokine rather than the odds of metastasis. Since we are utilizing HR with 95% CIs, this time we will employ a different template for formatting our data by selecting the second option in Figure 3A (notice the use of the extracted values directly without log transformation). As we are inputting HR with 95% CI, we will use the IOType “HRCI” in our function (see Table 2). Finally, as depicted in Figure 9, the number of studies included is relatively low (*n* = 4 studies); therefore, we will opt for a FEs model with “IV”.

## 5 META‐ANALYSIS OF PROPORTIONS AND RATES

### 5.1 Introduction to proportions and rates

A proportion is a measure that expresses the size of one part relative to the whole population while a ratio involves a relationship between two independent subsets of a population. Proportions often represented as a fraction or percentage (e.g., incidence and prevalence).^16^ Unlike OR and RR, proportions involve a single group rather than comparative pairwise analysis (e.g., two groups). As depicted in Figure 1, the majority of meta‐analyzes utilizing MetaXL primarily focus on estimating a pooled prevalence, indicating that MetaXL is both easy to use and reliable for this type of analysis. Prevalence always has a value between zero and one.

Two problems occur when pooling prevalence proportions.^4^ First is that the 95% CI can cross zero and one. The second is the because of the way it is pooled, when the proportion is at the extreme limits (e.g., near zero and one), the variance will approach these limits. Therefore, data transformation is required. By default, MetaXL uses double arcsin transformation, which is the recommended method by the developers,^17^ and it can be easily changes in the options settings (Figure 2 label C).

Rates are a function of time usually using the unit of person‐time at risk. Rate ratios represent the division of the incidence rate in an exposed group by the incidence rate in an unexposed comparison group (two groups).^18^ Conversely, rate difference denotes the disparity between two rates; for instance, it indicates the variance in incidence rate between a population group exposed to a causal factor and one not exposed to the factor.^19^ Table 3 displays the IOType codes relevant to pooling proportions, rates and related single‐group designs.

**Table 3:** IOType codes relevant to proportions and rates.

IOType	Effect size	Choice in input templates	Required data
Prev	Prevalence	Prevalence numbers (also choose number of categories)	Number of cases and total population size
NumRate	Rate	Rate numbers	Person time at risk (or new cases) and total population size
RateSE	Rate	Rate and standard error	Rate and standard error
Rateratio	Rate ratio	Person time and cases	Person‐time and event numbers (two groups)
Ratedif	Differences in rates	Person time and cases	Person‐time and event numbers (two groups)

### 5.2 Example 4: Using prevalence with multiple categories

In this example, we aim to estimate the prevalence of the pyramidalis muscle. This muscle can be present bilaterally (on both sides of the body), unilaterally (on one side of the body), or absent bilaterally. The data utilized in this example are modified and extracted from a study that conducted a meta‐analysis on anatomical variations of this muscle.^20^ Since we have three categories (bilateral, unilateral, and absent), we will select “Prevalence numbers” in the input templates (option number 7 shown in Figure 3A) and set the number of categories to three.

**Figure 10:**
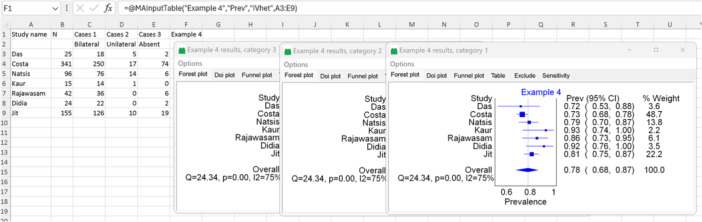
Shows the data and function used with the output forest plot for example 4.

As depicted in the template, we need to extract the prevalence as a proportion, utilizing studies that provided the number of cases in each category and the total population number (N represents the total). This time, we will manually edit the template by adding a secondary label for each category name to avoid confusion. For the IOType code, we will use “Prev” as we are pooling the prevalence. In this example, we will try setting “IVhet” as our model. Figure 10 showcases the data used, the Excel function, and the output forest plot for this example. MetaXL will provide the analysis for each of the three categories separately, as demonstrated in Figure 10.

### 5.3 Example 5: Incidence rate ratio and difference

In this fifth example, we aim to pool the ratio between the incidence rate of herpes zoster in individuals with inflammatory bowel disease and the incidence rate of herpes zoster in the general population (incidence rate ratio). The data are sourced from a meta‐analysis that contained five studies, presenting their extracted data in a well‐organized table and incorporating quality scores using the Newcastle‐Ottawa scale in their supplementary material.^21^ Given the limited number of included studies and the availability of numerical quality scores, we opt for the “QE” model.

Since our focus is on a rate ratio as the effect size of interest, we select “Person time and cases” from the input template menu (option number 10 shown in Figure 3A), while ensuring to check the “add quality field” option. We manually included a secondary label to aid in data formatting. As indicated in the template, we require the person‐time‐at‐risk (Ptime) and the number of herpes zoster cases in both groups (inflammatory bowel disease and healthy groups). During this step, it is crucial to ensure that all units are consistent (e.g., person‐years per 1000). Additionally, if the original study provided the incidence rate per 1000 person‐years, we can use it along with the number of cases to calculate the person‐year‐at‐risk per 1000.

**Figure 11:**
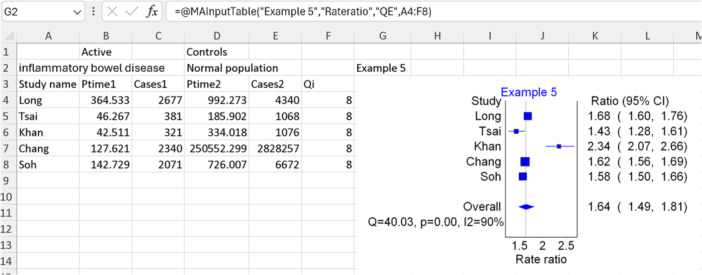
Shows the data and function used as well as the output forest plot for example 5.

From Table 3, we can see that the IOType section of the function can be populated with “Rateratio”. It is important to note that obtaining the differences in ratios necessitates the same inputs as rate ratios. Therefore, we can easily replace “Rateratio” with “Ratedif” to obtain the differences in ratios. Figure 11 illustrates the data utilized, the Excel function applied, and the resulting forest plot output for this fifth example.

## 6 META‐ANALYSIS OF MEANS

### 6.1 Introduction to means in meta‐analysis

The mean serves as a measure of central tendency, representing the average value within a set of numbers. When comparing means between two groups, we assess the difference in their respective averages. In meta‐analysis, we can pool two measurements of mean differences: weighted mean difference (WMD) and standardized mean difference (SMD).^22^ The distinction lies in the data given by the studies included. If all means within the studies share the same unit, utilizing WMD suffices. However, if different units are present, standardizing the mean (using SMD) becomes necessary, achieved by dividing the means by their respective standard deviations (SDs). MetaXL offers three equations for this process: Cohen's d (which divides by the pooled population SD), Hedges’ adjusted g (utilizing a correction factor for the sample SD), and Glass's Delta (relying only on the SD of the control group).

In MetaXL, we can input the requisite raw data for calculating mean difference (WMD or SMD), including population size, mean, and standard deviation. Alternatively, we can provide the mean difference (WMD or SMD) alongside its standard errors or 95% CIs. Table 4 outlines the relevant IOType codes for pooling mean differences.

**Table 4:** IOType codes relevant to mean differences.

IOType	Effect size	Choice in input templates	Required data
WMD	Weighted mean difference	Continuous numbers	Population size, mean, and standard deviation
Cohen	Standardized mean difference (using Cohen's d)	Continuous numbers	Population size, mean, and standard deviation
Hedges	Standardized mean difference (using Hedges’ adjusted g)	Continuous numbers	Population size, mean, and standard deviation
Glass	Standardized mean difference (using Glass's Delta)	Continuous numbers	Population size, mean, and standard deviation
ContCI	Any mean difference measurement (weighted, Cohen, Hedges or Glass)	Continuous and confidence interval	Mean difference measurement with its confidence interval
ContSE	Any mean difference measurement (weighted, Cohen, Hedges or Glass)	Continuous and standard error	Mean difference measurement with its standard error

### 6.2 Example 6: Using WMD

**Figure 12:**
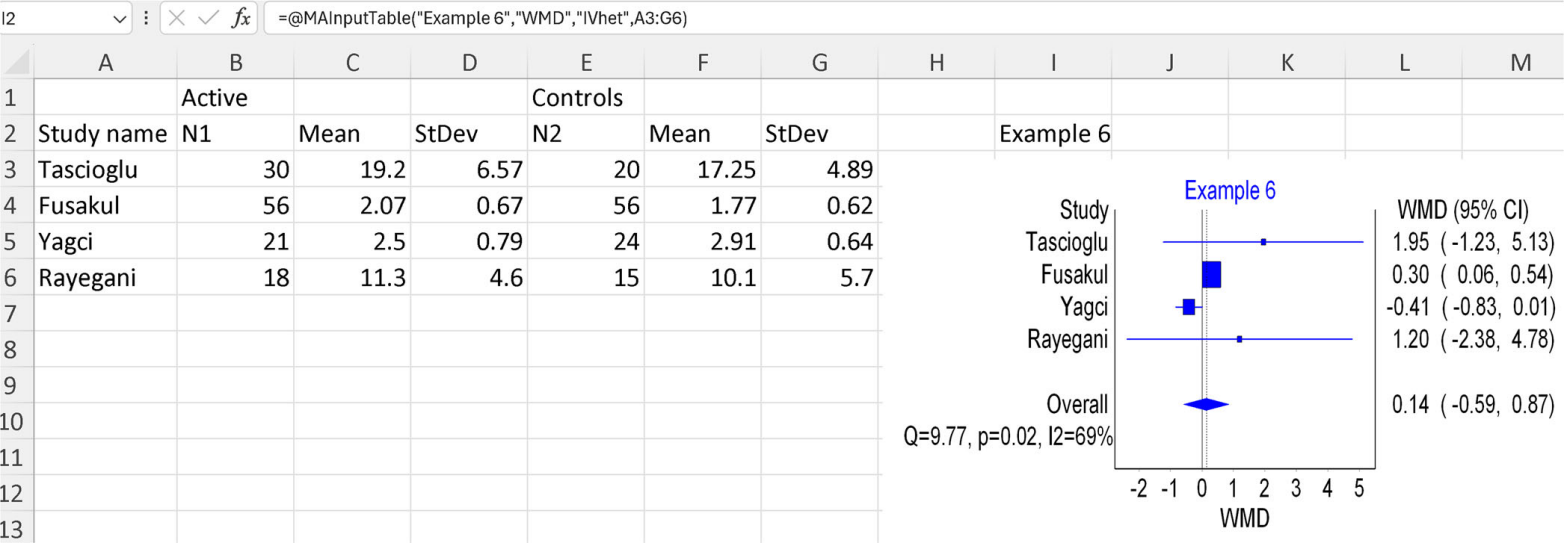
Shows the data and function used with the output forest plot for example 6.

In this example, we will use a modified version of data provided from a meta‐analysis investigating the impact of new laser therapy on carpal tunnel syndrome compared to a placebo.^23^ Assuming that the effect was assessed using the functional severity scale in all the included studies (e.g., same unit), we will use the WMD. The primary studies provided population size, mean, and standard deviation in both comparison groups. To insert the correct template, we will opt for the “Continuous numbers” (option number four in Figure 3A). Since we are employing mean difference measurement while providing the population size, mean, and standard deviation, we will set the IOType to “WMD”. Additionally, we will designate the “IVhet” as our model for this meta‐analysis. Figure 12 illustrates the modified data extracted, the Excel function used, and the resulting forest plot output for this example.

### 6.3 Example 7: Using SMD

**Figure 13:**
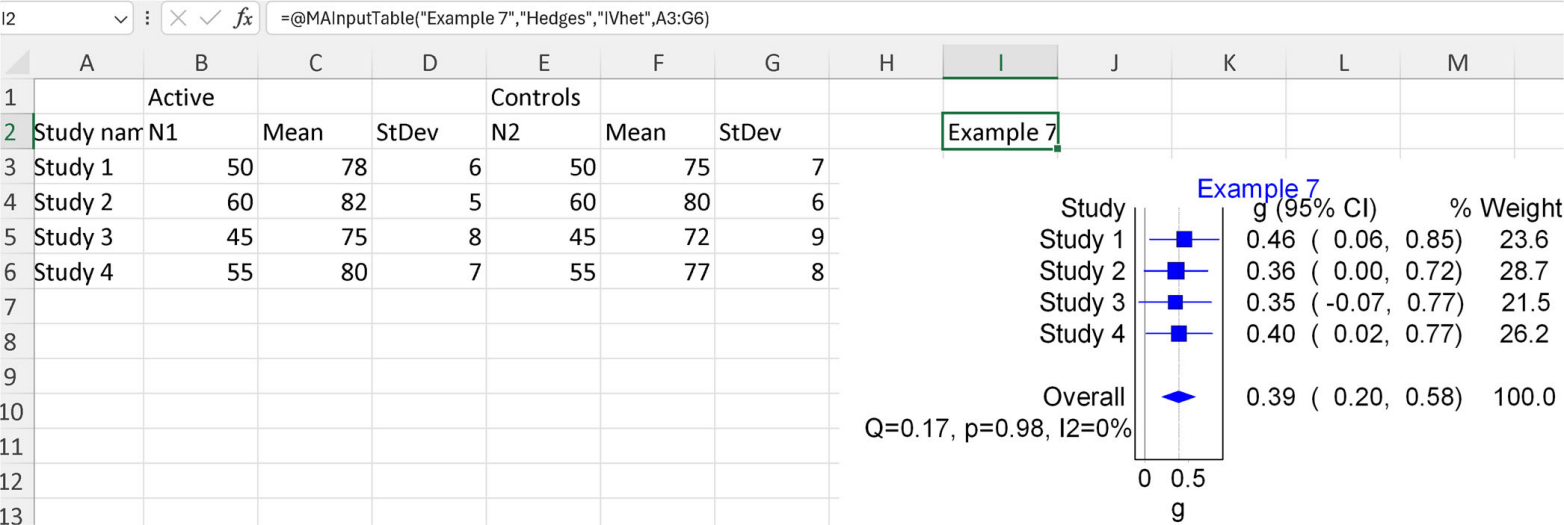
Shows the fictional data and function used with the output forest plot for example 7.

In this example, we will use fictional data to conduct a meta‐analysis comparing the effectiveness of two different teaching methods in improving student performance across various subjects. Each of the four included studies employed a different measure of student performance: test scores in mathematics, grades in science, self‐reported understanding of literature concepts, and teacher evaluations of writing skills. Therefore, we will utilize the population size, mean, and standard deviation to pool the SMD. For this example, we will employ Hedges’ adjusted g by selecting “Hedges” as the IOType. We will maintain consistency in data formatting and the meta‐analysis model by using the same template and model as in example 6. Figure 13 illustrates the modified data extracted, the Excel function used, and the resulting forest plot output for this example.

## 7 NETWORK META‐ANALYSIS (NMA)

### 7.1 Introduction to NMA

NMA is an extension of traditional meta‐analysis, allowing for the simultaneous evaluation of multiple treatments.^24^ It integrates both direct evidence from randomized control trials and indirect evidence from studies with shared comparators. Direct evidence arises from head‐to‐head comparisons between treatments, while indirect evidence derives from comparisons through common comparators.^24^ For example, we can combine data from studies comparing treatment A versus treatment B (direct evidence) and data from studies that compared either treatment A or treatment B versus placebo (indirect evidence). By synthesizing both types of evidence, NMA provides a comprehensive assessment of treatment effects across a network of interventions.

### 7.2 NMA assumptions

To pool these direct and indirect evidence, we need to make sure that the included studies have similar characteristics. To do that, we need to understand three key concepts in NMA, namely: homogeneity, transitivity and inconsistency.^25^

Homogeneity pertains to the consistency of trials within each pairwise comparison across the network. Transitivity is the validity of indirect comparisons and can only be assessed conceptually within each closed loop. It occurs when the common intervention (e.g., placebo) differs systematically between trials. This assumption is the sole requisite to performing NMA using MetaXL. The inconsistency in NMA refers to the same concept of heterogeneity meta‐analysis, but this time it includes evaluating direct, indirect, sources of indirect evidence. Inconsistency can be quantified in MetaXL by $\overset{®}{H}$, which is weighted average of H derived from Cochran Q ($\overset{®}{H}$<3 minimal inconsistency, 3<$\overset{®}{H}$<6 moderate inconsistency, $\overset{®}{H}$>6 high inconsistency).^26^

### 7.3 NMA in MetaXL

MetaXL employs the generalized pairwise modeling framework for conducting NMA.^26^ This approach entails repeatedly applying adjusted indirect comparisons. This procedure requires selecting one group (usually placebo or control group) as the common node in the network. In MetaXL, there is a separate Excel function for NMA known as the “MANetwork” function. It follows the format: =@MANetwork(Name, control, Table), where “Name” is a unique identifier set by the reviewer for the function, “control” is the name of the common node in the network, and “Table” is a group of Excel cells containing “MAInputTable” functions used for each comparison pooled separately, with two extra columns for the comparison groups in each “MAInputTable”.

### 7.4 Example 8: NMA

For this example, we aim to determine the most effective vaccine type against coronavirus disease (COVID‐19). We will utilize a modified version of the data inspired from a study that investigated this topic,^27^ enhancing it by adding fractional direct comparison studies to close the network loop and enhance educational comparisons. Additionally, we will restrict the vaccines to three types: viral vector, inactivated, and recombinant protein. Our analysis will involve performing a NMA of OR using the number of confirmed severe COVID‐19 cases after vaccination and placebo as a surrogate for vaccine efficacy. Table 5 displays the fractional raw data used in this analysis.

**Table 5:** Fractional raw data used for example 8 to conduct NMA with closed loop.

Study ID	Treatment group	Comparison group	Treatment group cases of severe COVID‐19	Total treatment group population	Comparison group cases of severe COVID‐19	Total comparison group population
Study 1	Viral vector	Placebo	18	4116	54	3113
Study 2	Viral vector	Placebo	22	4245	60	3287
Study 3	Viral vector	Placebo	20	4111	50	3786
Study 4	Viral vector	Placebo	25	4356	55	3369
Study 5	Inactivated	Placebo	15	3987	40	2567
Study 6	Inactivated	Placebo	18	3256	45	2755
Study 7	Inactivated	Placebo	17	3134	42	2654
Study 8	Recombinant	Placebo	12	2824	35	2256
Study 9	Recombinant	Placebo	14	2922	38	2344
Study 10	Recombinant	Placebo	16	3112	40	2465
Study 11	Recombinant	Placebo	10	2602	30	2546
Study 12	Viral vector	Recombinant	20	3904	28	2145
Study 13	Viral vector	Recombinant	22	4543	30	2223
Study 14	Viral vector	Inactivated	16	3523	22	1943
Study 15	Viral vector	Inactivated	18	3667	25	2133

**Figure 14:**
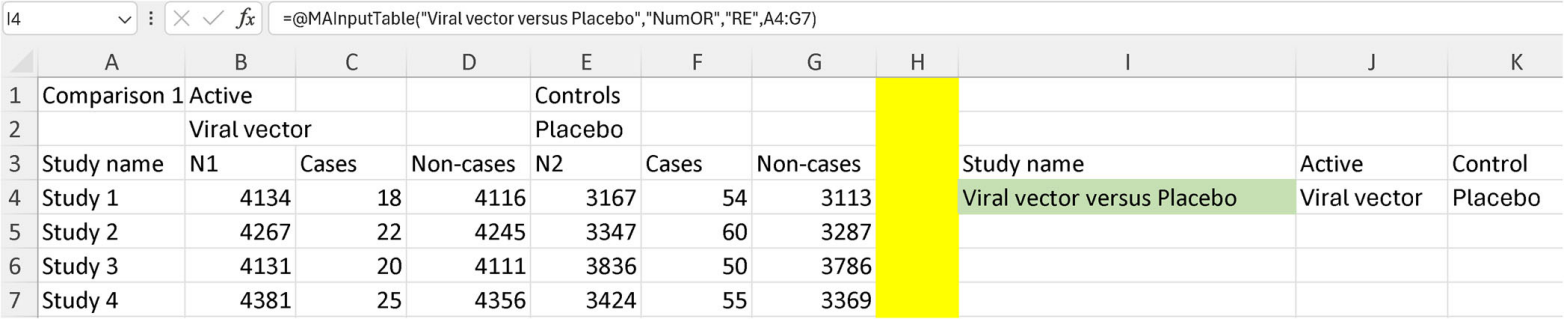
Shows the first “MAInputTable” function on the first comparison, which is on viral vector versus placebo.

We have five different comparisons in the included study: viral vector versus placebo, inactivated versus placebo, recombinant versus placebo, viral vector versus recombinant, and viral vector versus inactivated. Therefore, the first step in the analysis is to pool each comparison separately using “MAInputTable” functions, similar to what we did in the first two examples (using the same data templates). Since we are using binary ORs, we will use “NumOR” as shown in Table 2. For the analysis model, we will use “IVhet” as it is the only recommended method for NMA under the generalized pairwise modeling framework. We will add secondary labels to the template, and we will name the function based on the comparison group names. This time, instead of placing the “MAInputTable” functions in random Excel cells, we will first select the template for NMA (option number 12 in Figure 3A), and then input the “MAInputTable” functions below the “Study name” column. Figure 14 illustrates this step for the first “MAInputTable” function on the first comparison (viral vector vs. placebo).

**Figure 15:**
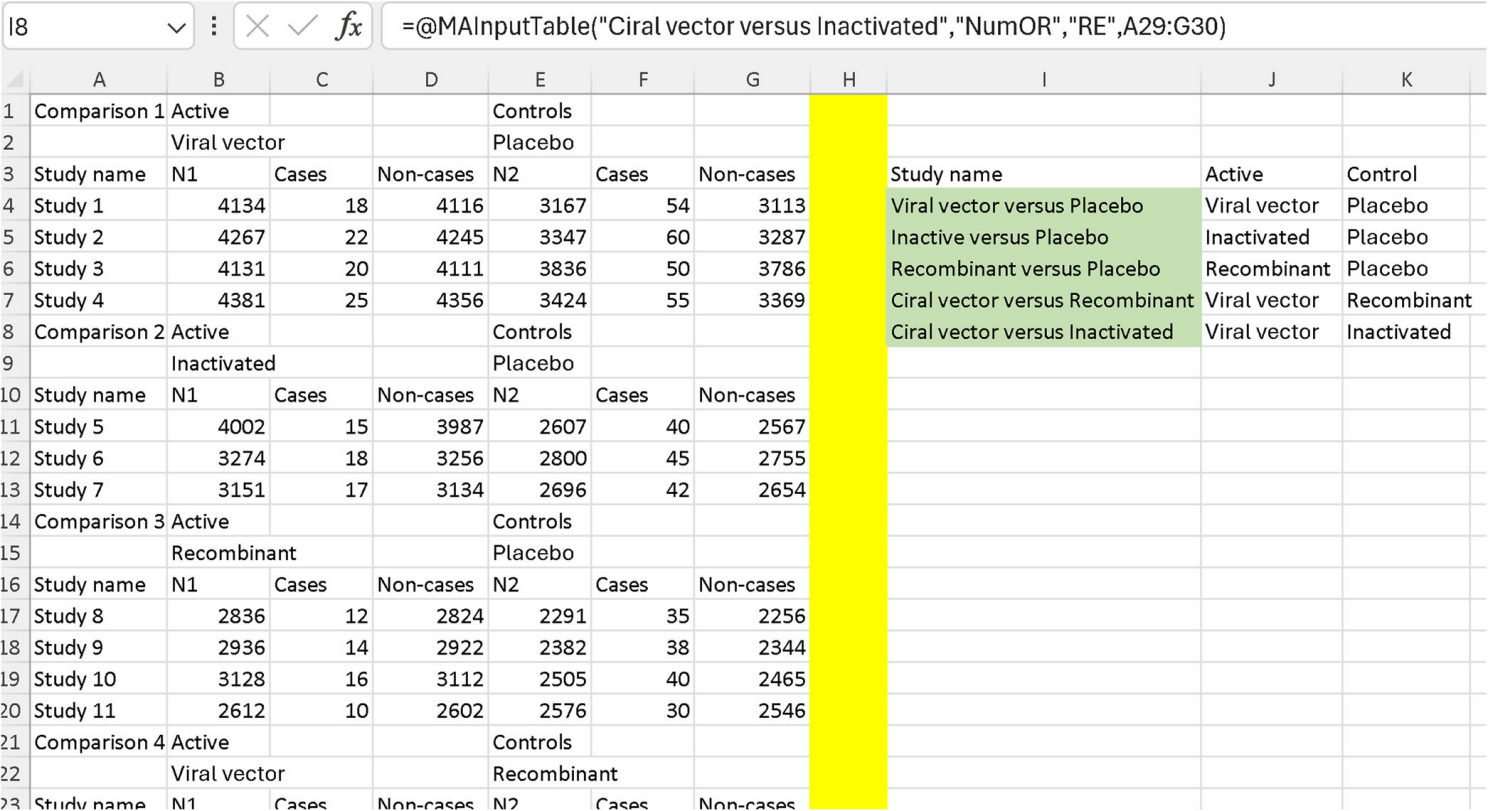
Displays complete “MAInputTable” functions for the comparisons made in the included studies, with cells containing “MAInputTable” functions highlighted in bright green.

We will now repeat the process four more times for the remaining four comparisons. Then, we will fill in the “Active” and “Control” columns as shown in Figure 14 on the right. The resulting complete table is displayed in Figure 15.

**Figure 16:**
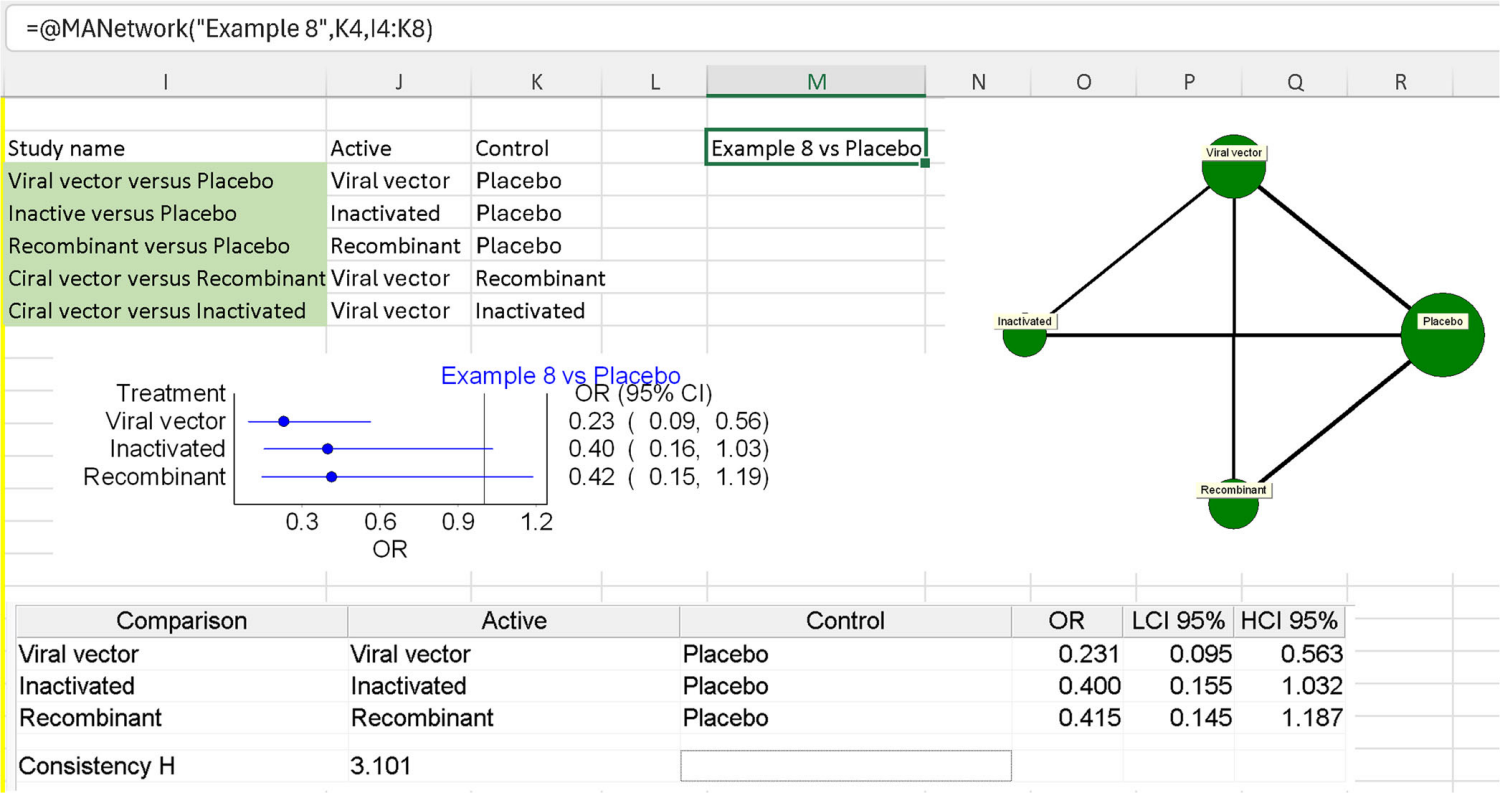
Shows the input, Excel function and results of the NMA performed in example 8.

Now, we can utilize the “MANetwork” function. We will name it “Example 8”, select any cell with the word “Placebo” in the table as the common node, and choose the three new columns as our table (excluding the table headers). The final function will be “= @MANetwork(“Example 8”, K4, I4:K8)”, as depicted in Figure 16, along with the resulting graphs from the analysis. It is important to note that the effect sizes for each treatment are used for the final comparison between them to determine which is better, and that $\overset{®}{H}$ is used to measure the inconsistency.

## 8 CONCLUSION AND FINAL REMARKS

MetaXL, a free and user‐friendly Excel add‐in, offers a plethora of options for conducting meta‐analysis across various effect sizes. However, researchers can streamline the meta‐analysis process by starting before the systematic review, ensuring they grasp the fundamentals of data formatting. This knowledge helps identify crucial data for analysis and its required format, minimizing the need for revisiting data extraction. Visiting MetaXL's data templates before the start can aid in this endeavor. Furthermore, understanding the desired effect size and its availability in the included studies is essential.

MetaXL simplifies the meta‐analysis process with a straightforward structure: input data into a template and populate the “MAInputTable” function. Attention to function formatting is crucial to avoid analysis errors. While researchers may encounter decision‐making scenarios, a grasp of the concepts and recommendations discussed herein should suffice. Lastly, the MetaXL user guide serves as a valuable resource for reference and additional examples whenever uncertainties arise.

Increased accessibility to meta‐analysis methods through free software has the potential to enhance the quality and reach of clinical research. However, there is a growing concern about the potential for research waste if these tools are not used properly, as highlighted by one of the peer reviewers. Research waste includes not only poorly conducted studies, but also unnecessary research, unpublished projects, and research published without sufficient justification or relevance.^28^ Nowadays, the publication rate for meta‐analyzes is rising, corresponding the increase seen in studies collecting primary data. Unfortunately, many of these publications lack value and fail to advance scientific knowledge effectively.

A significant issue is that researchers without proper training in clinical epidemiology often produce poorly designed or repetitive investigations. Without optimizing the application of clinical epidemiology principles to conduct, appraise, or apply clinical research, especially in critical areas like diagnosis or treatment, their output can become research waste.^28^ Methods of meta‐analysis should be aligned with the key requirements of addressing relevant research questions, using good data, and involving clinicians with topic‐specific knowledge in the research process.

To avoid contributing to research waste, as recommended by previous literature,^28^ a meta‐analysis must address a relevant research question based on a gap in knowledge, employ robust epidemiological and statistical methods, include both content experts and methodologists, and provide a clear rationale for conducting a new meta‐analysis if previous ones on the same topic exist, referencing and discussing them thoroughly.

## AUTHOR CONTRIBUTIONS

**Ibrahim Elmakaty**: Conceptualization, methodology, validation, investigation, writing—original draft, writing—review & editing, supervision, project administration, figures and tables, and content organization.

## CONFLICT OF INTEREST STATEMENT

The author discloses that they were a medical student at the same university as Suhail Doi, one of the developers of MetaXL, and worked on a projects under his supervision. The author declares no conflict of interest.

## ETHICS STATEMENT

Not applicable.

## Supporting information

Supporting information.

## Data Availability

The data that supports the findings of this study are available in the supplementary material of this article.
